# The Cut-off Values for the Diagnosis of Hamstring Shortness and Related Factors

**DOI:** 10.4274/balkanmedj.2017.1517

**Published:** 2018-09-21

**Authors:** Muhammed Şeref Yıldırım, Filiz Tuna, Derya Demirbağ Kabayel, Necdet Süt

**Affiliations:** 1 Department of Physical Therapy and Rehabilitation, Trakya University School of Health Science, Edirne, Turkey; 2 Department of Physical Medicine and Rehabilitation, Trakya University School of Medicine, Edirne, Turkey; 3 Department of Biostatistics, Trakya University School of Medicine, Edirne, Turkey

**Keywords:** Flexibility, hamstring muscles, joints, knee, range of motion

## Abstract

**Background:**

Hamstrings are one of the most frequently evaluated muscle groups for flexibility in the lower extremity. Passive and active knee extension angle values are used as an indirect indicator of hamstring flexibility. However, the lack of consensus on the cut-off values leads to the use of inconsistent angle values in determining the hamstring tightness.

**Aims:**

To establish the normative and cut-off values of the passive and active knee extension angles for healthy young adults and to determine the associated factors including the quadriceps strength.

**Study Design:**

A cross-sectional study.

**Methods:**

A total of 123 volunteer university students, aged 18-24 years, who met the inclusion criteria were included in this study. Passive and active knee extension assessments of the subjects were performed. Subsequently, on the next day, both knee extensor concentric muscle strength of the participants was measured in the isokinetic system. The knee extension angles and the knee extensor strength were recorded as the mean values of the right and the left sides.

**Results:**

Passive knee extension angles of 17.1°±9.1° and 9.8°±5.7° and active knee extension angles of 17.8°±9.1° and 13.4°±6° were described as normative values in men and women, respectively. The cut-off values for the diagnosis of hamstring shortness were as follows: passive knee extension angle >32.2° for males and >19.2° for females and active knee extension angle >33.0° for males and >23.4° for females. A significant positive correlation was observed between knee extension angles and isokinetic knee extensor muscle strength in all participants. The knee extension angle and hamstring flexibility were not affected by dominance.

**Conclusion:**

The knee extension angles of healthy young people seem to be lower than the results currently reported in the literature. There s a positive correlation between knee extension angles and isokinetic knee extensor muscle strength.

Flexibility, defined as the rate of muscle tissue lengthening, is one of the important components in maintaining physical fitness (1). Inadequate muscle flexibility can lead to problems related to changes in lower extremity biomechanics. Tight hamstrings affects posture, range of motion of lower limbs (2), and gait pattern (3). Plantar fasciitis (4), Patellofemoral Pain syndrome (5), and low back pain (6) have also been reported to be associated with hamstring tightness. Methods for measuring hamstring flexibility include straight leg raise, sit-and-reach, toe-touch, and knee extension angle tests (active and passive) (7). Although the first three methods are often used, their results are affected by trunk and hip flexibility. Active knee extension (AKE) (8) and passive knee extension (PKE) (9) tests allow for more isolated hamstring evaluations by stabilizing the hip joint. The passive version of AKE (PKE) was designed by claiming that AKE test results depend on the subject’s quadriceps strength (9). However, to our knowledge, there is no study that has examined the relationship between quadriceps strength and knee extension angles in the literature. Although the active method is easier as there is no need for a second examiner, several researchers prefer the passive method because of the potential effect of quadriceps strength on AKE values. Although AKE and PKE tests are often used in current studies (4,6), there are few studies that report the tests’ normative values. As far as we know, there are only two studies determining the normative values of AKE (10) and PKE (11), and no study has yet reported the cut-off values. The angle values reported by the studies using AKE and PKE tests in evaluating hamstring flexibility are inconsistent (Table 6). The lack of cut-off values leads to the use of inconsistent angle values in determining the hamstring tightness. These inconsistent cut-off values vary over a wide range of 15°-60° (2,12,13). When we consider the age-related changes in flexibility, it appears that age-specific reference and cut-off values are necessary (14). Research on the factors affecting hamstring flexibility seems to focus on age and gender. The effects of dominance and knee muscle strength on the hamstring flexibility are not well known due to inadequate research on this subject. Therefore, the aims of our study were to establish the normative and cut-off values of passive and AKE angles for healthy young adults and to determine the associated factors. We also aimed to reveal whether the values of the AKE results were affected by quadriceps strength.

## MATERIALS AND METHODS

This study was carried out in Trakya University, School of Health Sciences. The study was approved by the Ethics Commission of Non-invasive Clinical Trials of Trakya University (approval number: 2016/220).

### Participants

All subjects signed the informed consent prior to participating in the study, and the rights of these subjects were protected. One hundred and thirty-nine individuals aged between 18 and 24 years were evaluated. The inclusion criteria were a) no generalized joint hypermobility, b) a body mass index (BMI) of <30 kg/m^2^, c) no recent history of hamstring strain, and d) no known history of hip or knee joint disease. According to these criteria, 14 subjects were excluded due to generalized joint hypermobility and 2 subjects were excluded due to obesity. Finally, 123 healthy university students (62 females, 61 males) were included in this study.

### Procedures

The height and weight of the subjects were recorded using a standardized medical scale. Age and past medical history were recorded using a questionnaire. The dominant leg was also selected by asking the subjects about their preferential leg for kicking a ball. Then, we collected the data basically in the following two steps: 1) indirect measurements of hamstring muscle length (HML) and 2) isokinetic measurements for knee extensors.

Isokinetic measurements were performed a day after indirect muscle length measurement.

### Indirect measurements of hamstring muscle length

Indirect HML was assessed by a physician and a physiotherapist by measuring the AKE and the PKE angles. Before testing, all participants warmed up for 3 min at average intensity on a pedal ergometer without resistance (MSD Oxycycle 3, USA) to standardize the amount of activity. The same initial position was used for active and passive measurements of the knee extension angle. Therefore, these tests were performed consecutively. The PKE test was performed prior to the AKE test assuming that the AKE test would cause reciprocal inhibition in the hamstrings (15). A standard, universal goniometer was used to determine the knee motion degree during the knee extension test. A wooden box measuring 45 cm wide, 42 cm high, and 25 cm deep was also used to place the hip in the initial position of the test. The box was secured to the table using a velcro strap.

### Initial position of the tests

Individuals were positioned in supine with the contralateral extremity in extension. The ipsilateral hip and the knee were flexed to 90° flexion with the ischial tuberosity placed against the box. Four straps were used for stabilization as follows: the first strap was used to stabilize the box, a second strap was used to secure the subject’s contralateral thigh, a third strap around the ipsilateral thigh was used to minimize the hip flexion, and the fourth strap on the subject’s iliac anterior spines was used to minimize the posterior pelvic tilt during the test. The subjects were instructed to maintain the pelvic tilt and not to separate the thighs from the box until the end of the test. The mean AKE and PKE angles calculated for the right and the left extremities were recorded (average of three measurements). To prevent bias, the goniometer dial was covered with a paper.

### PKE test

After taking the initial position, the subject’s ipsilateral knee was passively straightened to a point where the subject reported a strong but tolerable stretch in their hamstring. The PKE angle was then measured by the second examiner using the goniometer (9). The hip angle determined as 120° in the original study (9) was used as 90° because of feasibility. Similarly, in the current literature, it is preferred to use a 90° hip angle (7,16,17).

### AKE test

After taking the same initial position, the subject was asked to actively extend the ipsilateral knee with the foot relaxed in the plantar flexion. Knee extension stretched the hamstring muscles until myoclonus occurred. The myoclonus consisted of contraction and relaxation of the hamstrings and the quadriceps femoris. The subject was then told to slightly flex the knee till the myoclonus stopped. At this point, the degree of knee flexion was recorded  (8). The recorded PKE and AKE angle values were calculated according to normal distribution, and the cut-off values were determined for hamstring shortening. The AKE and the PKE angle values were also classified according to dominance.

### Isokinetic measurements

Isokinetic measurements of the subjects were performed one day after the knee angle measurements. Subjects were tested by CSMI Cybex HUMAC/NORM, USA, isokinetic tester with the model number 502140. Each participant performed a 5 min warm-up in a bicycle ergometer and then seated upright with their arms folded across the chest. The subjects were secured using backrest support and velcro straps. Before the testing protocol, the subjects were allowed to try three submaximal isokinetic concentric knee extension and flexion repetitions at 60°/s and four repetitions at 240°/s. After familiarization, the subjects were instructed to give full effort and received verbal encouragement during testing. They were given 4 maximal contractions at 60°/s and 15 maximal contractions at 240°/s for each testing set. Peak torque and total work of both knee flexors and extensor muscle groups were calculated (18).

### Statistical analysis

Descriptive statistics were expressed as mean ± standard deviation. All variables were tested for normal distribution using the Kolmogorov-Smirnov test. Statistical significance was accepted at p<0.05. Age, weight, height, and BMI values according to gender were tested using Student’s t-tests. The association between knee extension angles and isokinetic parameters was examined using the Mann-Whitney U test. The Wilcoxon signed rank test was used to compare the differences in the participants’ dominant and nondominant knee extension angles. The association between PKE and AKE angles and isokinetic parameters was examined using the Spearman correlation analysis. All statistical analyses were performed using the Statistical Package for the Social Sciences (SPSS version 20.0).

## RESULTS

Subjects’ mean age, height, weight, and BMI were calculated and categorized according to males, females, and total groups (Table 1). The mean values of AKE and PKE for the right side, the left side, and both sides combined were calculated. The normative values determined in this study were as follows: PKE angle 17.1°±9.1° for males and 9.8°±5.7° for females and AKE angle 17.8°±9.1° for males and 13.4°±6° for females (Table 2). The cut-off values of the AKE and the PKE test for the detection of short hamstring muscle flexibility were calculated based on the normal distribution approach (95% of the population was accepted as normal). The cut-off values were as follows: PKE angle >32.2° for males and >19.2° for females and AKE angle >33.0° for males and >23.4° for females (Table 3). A majority of participants (88.6%) reported that their right leg was dominant, whereas the remaining subjects reporting left leg dominance. We found no significant differences in the mean AKE and PKE angles between the dominant and the nondominant sides (Table 4). A significant correlation was observed between the knee extension angles and the isokinetic quadriceps strength (peak torque-0°/sn) in all participants (p<0.05) (Table 5). However, when male and female participants were evaluated separately, no significant relationship was observed between the same parameters (p>0.05) (Table 5).

## DISCUSSION

A review of the past literature shows that there is a lack of reference values determined in terms of knee extension angles to identify hamstring shortening in healthy young people. In the study conducted by Erkula et al. (2) in healthy adults, subjects with a knee extension angle <60° were included in the hamstring shortening group. The authors determined the angle 60° by adding 5°-10° to the knee extension angle shortness limit that shows pathological implication for children (19). Some researchers (12,13) used the values 15° and 20° as the cut-off for hamstring shortening based on their clinical experience. Due to the lack of investigation in this area, we aimed to determine the normative values for PKE and AKE angles among university students in this study. The aim of our study was to contribute to the literature by determining the PKE and AKE angle cut-off values required to define hamstring shortening. We investigated the relationship between the PKE and AKE angle values and the concentric isokinetic knee muscle strength parameters. Our results showed that the knee extension angles of healthy young people were lower than the results currently reported in the literature (Table 6) (10,11). The factors that influenced these differences are suggested to be the assessment method, the endpoint considered during the test, the implication of the preassessment warm-up period, and the age groups. Regarding the evaluation method, we used a wooden box that is in contact with the back of the thigh for hip stabilization as used by Kuilart et al. (13). On the other hand, Corkery et al. (10) used PVC pipes in contact with the front thigh to stabilize the hip at 90° during the measurement. Since the average AKE values obtained from people with the hamstring shortening (AKE >15°) in the study of Kuilart et al. (13) were similar to the AKE normative values obtained from healthy college students by Corkery et al. (10) the method used for hip stabilization is considered to be effective in the measurement results (Table 6). There is also a study using an inclinometer placed on the thigh to stabilize the hip at 90° (7). In that study, Davis et al. (7) identified the endpoint as a strong but tolerable stretch on the hamstrings. The results of our study are similar to those of Davis et al. (7) in terms of the age group we examined, the endpoint we used, and the PKE angle we obtained. Davis et al. (7) had presented the obtained PKE angle values as the angle that the tibia makes with the horizontal plane, that is, the “90-PKE” formula. Based on this formula, the results for men, women, and all participants were 71.6°±9.6°, 77.7°±9.5°, and 74.6°±10.0°, respectively. When these results were reflected as the PKE angle in our study, the values were determined as follows: 18.4° for men, 12.3° for women, and 15.4° for all participants (7). In the case of AKE angle measurement, there are studies using also the “first stretch sensation in hamstring” as the endpoint to the knee extension (10,11). In our evaluation, the participants were motivated to bring the knee to extension, until myoclonus was observed between the flexor and the extensor muscles. At the end of knee extension, the participant was instructed to move the knee to some degree of flexion to stop the myoclonus. The fact that our results are lower than the current research may be explained by the differences in determining the endpoint.

When the relationships among age, gender, and hamstring flexibility are considered, it has been reported that gender differences in terms of hamstring flexibility for the preadolescence period are not significant (19). In the young population that we evaluated in this study, the hamstring flexibility of women (AKE and PKE angle values) was significantly higher than that of men. This result is consistent with the data reported in the literature (10,11). We did not find any study investigating the effect of dominance on the knee extension angle in healthy young people. In our study, we found that the knee extension angle and the hamstring flexibility are not affected by dominance. Macedo and Magee (20) who investigated joint range of motions, found clinically insignificant small angle differences between dominant and nondominant sides. In addition, in athletes, the nondominant lower extremities have been reported to be more flexible (21,22). This is due to the more intense use of the dominant side by the athletes, the minimal injury on the dominant side, and the accumulation of scar tissue (20). To elucidate the relationship of flexibility between dominant and nondominant extremities, we believe that a study involving athletes and non-athletes is necessary. We did not find the cut-off values in the literature, which we determined for hamstring shortening of the age group that we examined. Most of the studies used the cut-off values that were obtained from the research based on the pediatric age group (2) or the cut-off values based on clinical experience (7,13) in the definition of hamstring shortenings. The cut-off values of the PKE and AKE angles in terms of determining the hamstring shortening in youth are as follows: 32.2° and 33.0° for males, 19.2° and 23.4° for females, and 27.3° and 28.9° for the general population. We recommend that the angle values over the specified numbers in young adults (aged 18-24 years) be evaluated as “hamstring shortness.” Kuilart et al. (13) rated the average AKE angle as 35.2° by evaluating individuals who were thought to have hamstring shortening (AKE angle >15°). Considering the cut-off values we determined, the AKE average of the hamstring shortening group in our study was calculated as 41.6° for men and 31.2° for women. This result was similar to the finding reported by Kuilart et al. (13) that was based on individuals with hamstring shortness.

Another purpose of our study was to disclaim the negative correlation between knee extension angles and isokinetic extensor muscle strength. We did not come across any study in the literature regarding this issue.

According to our results, in contrast to what some researchers have claimed, a positive correlation was found between hamstring flexibility (AKE) and isometric muscle strength (extensor group peak torque) (60°/s); hence, we can certainly state that the AKE test results do not decrease due to an increase in quadriceps strength, as claimed by Fredriksen et al. (9). We believe that quadriceps strength has no positive or negative effect on AKE test results. There are three findings that make us believe this way, which are as follows:

- There was also a positive correlation of the PKE angle values with the isometric knee extensor muscle strength (extensor group peak torque 60°/s).

- When male and female participants were evaluated separately, there was no significant relationship between hamstring flexibility and knee extensor muscle strength. This can be attributed to a reduction in the number of cases when the participants were grouped by gender.

- According to our results, knee muscle forces (peak torques) of dominant extremities were significantly higher than those of nondominant ones. Nonetheless, there was no significant difference between the AKE angles that belonged to the dominant and nondominant extremities.

In conclusion, the knee extension angles of healthy young people seem to be lower than the results currently reported in the literature. There is a positive correlation between extension angles and isokinetic knee extensor muscle strength.

## Figures and Tables

**Table 1:**
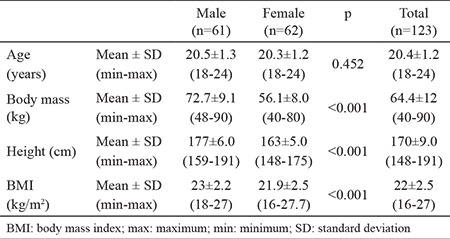
Means and standard deviations for age (years), weight (kg) and height (cm) of participants

**Table 2:**
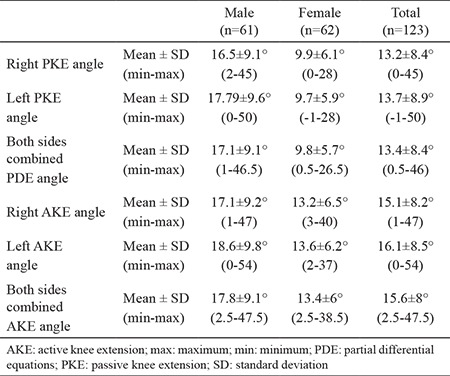
Means and standard deviations for AKE and PKE angles for right and left sides and the groups combined

**Table 3:**
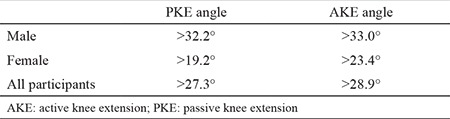
Cut-off values for passive knee extension and active knee extension angles in determining hamstring shortening

**Table 4:**
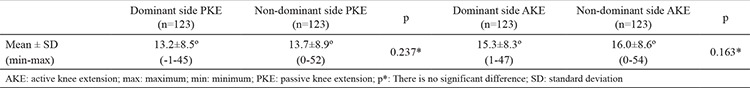
Comparison of dominant and nondominant knee extension angles

**Table 5:**
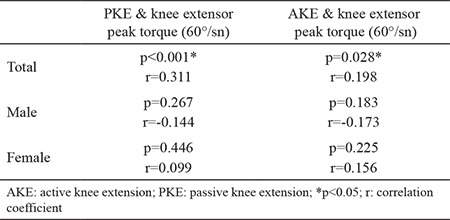
Relationship between knee extension angles and knee extensor muscle strength

**Table 6:**
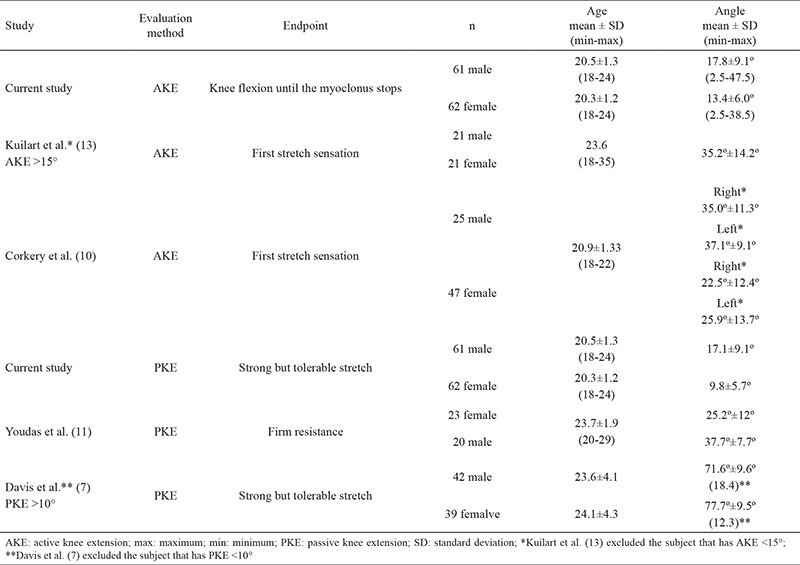
Summary of studies that estimated knee extension angle in healthy adults
